# The role of Beringia in human adaptation to Arctic conditions based on results of genomic studies of modern and ancient populations

**DOI:** 10.18699/VJGB-23-45

**Published:** 2023-07

**Authors:** B.A. Malyarchuk

**Affiliations:** Institute of Biological Problems of the North, Far-East Branch of the Russian Academy of Sciences, Magadan, Russia N.A. Shilo North-East Interdisciplinary Scientific Research Institute, Far-East Branch of the Russian Academy of Sciences, Magadan, Russia

**Keywords:** genomics, paleogenomics, mitochondrial DNA, Y chromosome, adaptation, Beringia, peopling of America, геномика, палеогеномика, митохондриальная ДНК, Y-хромосома, адаптация, Берингия, заселение Америки

## Abstract

The results of studies in Quaternary geology, archeology, paleoanthropology and human genetics demonstrate that the ancestors of Native Americans arrived in mid-latitude North America mainly along the Pacific Northwest Coast, but had previously inhabited the Arctic and during the last glacial maximum were in a refugium in Beringia, a land bridge connecting Eurasia and North America. The gene pool of Native Americans is represented by unique haplogroups of mitochondrial DNA and the Y chromosome, the evolutionary age of which ranges from 13 to 22 thousand years. The results of a paleogenomic analysis also show that during the last glacial maximum Beringia was populated by human groups that had arisen as a result of interaction between the most ancient Upper Paleolithic populations of Northern Eurasia and newcomer groups from East Asia. Approximately 20 thousand years ago the Beringian populations began to form, and the duration of their existence in relative isolation is estimated at about 5 thousand years. Thus, the adaptation of the Beringians to the Arctic conditions could have taken several millennia. The adaptation of Amerindian ancestors to high latitudes and cold climates is supported by genomic data showing that adaptive genetic variants in Native Americans are associated with various metabolic pathways: melanin production processes in the skin, hair and eyes, the functioning of the cardiovascular system, energy metabolism and immune response characteristics. Meanwhile, the analysis of the existing hypotheses about the selection of some genetic variants in the Beringian ancestors of the Amerindians in connection with adaptation to the Arctic conditions (for example, in the FADS, ACTN3, EDAR genes) shows the ambiguity of the testing results, which may be due to the loss of some traces of the “Beringian” adaptation in the gene pools of modern Native Americans. The most optimal strategy for further research seems to be the search for adaptive variant

## Abstract (ru)

Результаты исследований в области четвертичной геологии, археологии, палеоантропологии и генетики человека показывают, что предки американских индейцев прибыли в средние широты Северной Америки главным образом по тихоокеанскому побережью. Но до этого они населяли Арктику и во время последнего ледникового максимума укрывались в рефугиуме на территории Берингии, соединявшей Евразию и Северную Америку. Генофонд американских индейцев представлен уникальными гаплогруппами митохондриальной ДНК и Y-хромосомы, эволюционный возраст которых составляет от 13 до 22 тыс. лет. Результаты анализа палеогеномных данных также свидетельствуют о том, что во время последнего ледникового максимума на территории Берингии сохранялись группы населения, возникшие в результате взаимодействия древнейшего верхнепалеолитического населения Северной Евразии и пришлых групп населения из Восточной Азии. Примерно 20 тыс. лет назад началось формирование популяций Берингии, а время их существования в относительной изоляции оценивается примерно в 5 тыс. лет. Таким образом, период адаптации берингийцев к условиям Арктики мог занять несколько тысячелетий. Адаптация предков америндов к высоким широтам и холодному климату подтверждается геномными данными, показавшими, что адаптивные генетические варианты у американских индейцев ассоциируются с различными метаболическими пу- тями: процессами продукции меланина в коже, волосах и глазах, функционированием сердечно-сосудистой системы, энергетическим обменом и особенностями иммунного ответа. Между тем анализ существующих гипотез об отборе некоторых генетических вариантов у берингийских предков америндов в связи с адаптацией к условиям Арктики (например, в генах FADS, ACTN3, EDAR) демонстрирует неоднозначность результа- тов проверки, что может быть связано с утратой некоторых следов «берингийской» адаптации в генофондах современных американских индейцев. Наиболее оптимальной стратегией дальнейших исследований представляется поиск адаптивных вариантов с помощью анализа палеогеномных данных с территории Берингии, однако таких генетических данных пока очень мало.

## Introduction

Interdisciplinary research is important in examining issues related to the role of Beringia as an Arctic land bridge in the settlement of the Western Hemisphere. Unfortunately, the flooding of the lowlands of Beringia by rising sea levels 12,000 years ago led to the loss of a large number of archaeological sites, including possible human settlements. As a result, the main hypotheses associated with the history of the peopling of the Americas are based primarily on a synthesis of archaeological, geological, and biological data from the more accessible regions of Asia and the Americas (Potter et al., 2018; Batche- lor et al., 2019; Waters, 2019). According to one model of American settlement, ancient people from Northeast Asia migrated to the Americas via Beringia along an ice-free corridor in what is now Western Canada, which was created by melting glaciers at the end of the Pleistocene. According to another scenario, Native American ancestors actively developed the coastal zone and migrated to northwest North America along the southern coast of Beringia (Waters, 2019; Willerslev, Meltzer, 2021).

However, both of these scenarios appear to underestimate the more complex role that Beringia played in the settlement of the Americas by human populations (Potter et al., 2018). Results of new geochronological studies suggest that the “Canadian Corridor” was blocked by ice during presumed human migrations ~15–16 thousand years ago and was only freed ~13.8 thousand years ago (Clark et al., 2022). Therefore, it seems more likely that the migration route is the northwest coast of America (Lesnek et al., 2018). Since the melting of the glaciers in these territories could have occurred as early as 18,000 years ago, the coastal migration route could have been developed by the first Americans ~17,000 years ago (Lesnek et al., 2018).

At the same time, the results of genomic studies indicate that the ancestors of the Native Americans appear to have lived in the Beringian Arctic for quite a long time and survived the last glacial maximum there, which terminated ~19,000 years ago (Tamm et al., 2007; Amorim et al., 2017; Moreno-Mayar et al., 2018a; Niedbalski, Long, 2022). It is assumed that these populations adapted to the harsh conditions of the Far North while living in Beringia, and as the climate warmed, Beringian descendants migrated to Northwest America

## Population genomics of indigenous populations of Siberia: genetic origins of Native Americans

Due to the rapid development of DNA sequencing techniques, the genomic approach is widely used in modern research in archaeology and paleoanthropology. Genetic studies of the indigenous populations of Siberia have shown that the most ancient genetic components, which were present as early as 30–40 thousand years ago in the most ancient inhabitants of both southern and northern Siberia, were later lost as a result of genetic drift and population replacement periods. Thus, genomic analysis of the ancient populations of Siberia (in the time interval from 31 to 0.6 thousand years ago) has shown that in Siberia there were several periods of almost complete population replacements (Sikora et al., 2019). The most ancient Late Paleolithic population of Siberia was replaced 20–11 thousand years ago by newcomers from East Asia, resulting in the formation of Siberian populations that gave rise to the ancestors of both Paleosiberians and Native Americans. In the time interval from 11 to 4 thousand years ago, this population was partially replaced by other groups of East Asian origin, which gave rise to the modern populations of Siberia. Additional gene flows in both directions along the Bering Strait were also reported at this time. These flows affected the genetic structure of Eskimo and Na-Dene populations (Flegontov et al., 2019; Sikora et al., 2019).

The results of paleogenomic studies have shown that the formation of ancestral populations to ancient peoples of northern Siberia and America could have occurred in a wide area from the Trans-Baikal region to Beringia (Sikora et al., 2019; Yu et al., 2020; Kilinç et al., 2021). Paleogenomic data indicate that during the Holocene the populations of Northeast Asia (from the Cis-Baikal region to modern Yakutia) were very dynamic in contrast to the Trans-Baikal populations, which demonstrate genetic continuity from the Mesolithic to the Bronze Age (Kilinç et al., 2021). Evidence was also obtained that bearers of the Belkachin archaeological culture in Yakutia can be considered ancestors of the Paleo-Eskimos, who migrated to the Americas about 5–6 thousand years ago (Kilinç et al., 2021).

Population studies of polymorphism of mitochondrial DNA (mtDNA) and Y-chromosome, which are inherited through the maternal and paternal lineages, respectively, have developed greatly over the past 40 years. The results of molecular dating of the evolutionary age of the mtDNA and Y-chromosome haplogroups distributed among the modern indigenous populations of Siberia demonstrate that the most ancient genetic lineages in the south of Siberia belong to the postglacial period (younger than 21,000 years), while in the northeast of Siberia their evolutionary age does not exceed 10,000 years (Derenko et al., 2010, 2014; Malyarchuk et al., 2011; Duggan et al., 2013).

Genetic data for the present-day populations of Siberia are thus inconsistent with archaeological and paleoanthropological data showing the presence of Homo sapiens both in the south and in the north of Siberia as far back as 40–45 thousand years ago (Vasil’ev et al., 2002; Pitulko et al., 2004), but suggest that due to genetic drift, the most ancient genetic lineages were lost in the modern Siberian population (Derenko et al., 2010, 2014; Raghavan et al., 2014). This assumption was completely confirmed by the results of the paleogenomic study of the Siberians, which showed, as noted above, that in Siberia there have been several periods of almost complete replacements of the earlier inhabitants (Sikora et al., 2019). The genetic data obtained thus do not contradict the hypothesis that, during the last glacial maximum, the Arctic part of Asia and America (in particular, Beringia) could have been inhabited by humans

Based on the mtDNA variability data, it has been established that the divergence between the ancestors of the Siberian and Native American populations occurred ~25,000 years ago, the evolutionary age of Native American founding mtDNA lineages is approximately 18.5 thousand years, and their spread across Americas began ~16,000 years ago (Llamas et al., 2016). The mitochondrial gene pool of Native Americans is represented by only seven mtDNA haplogroups (A2, B2, C1, D1, D4h3a, C4c, and X2a), the evolutionary age of which ranges from 13 to 20 thousand years ago (Perego et al., 2009; Hooshiar Kashani et al., 2012). All of the Native American mtDNA haplogroups are unique, and for six of them (except D4h3a) there are still no analogues found anywhere else in the world, which means that these haplogroups were formed in deep isolation and, apparently, while living in Beringia

For the haplogroup D4h3a, a related D4h3b-haplotype was found in modern Chinese, which diverged from the common D4h3 ancestor by ~18.3 thousand years, and the evolutionary age of D4h3a itself is ~13 thousand years (Behar et al., 2012). It is also noteworthy that in all cases (except X2a) the Native American mtDNA haplogroups are derived from East Asian ancestors within haplogroups A, B, C, and D, predominantly distributed in East Asia. Meanwhile, the area of haplogroup X is located in Western Eurasia, and therefore the appearance of haplogroup X2a in the Beringians (and thus in the ancestors of the Amerindians) could have happened on the basis of the gene pool of their Upper Paleolithic ancestors, related to the population of Western Eurasia. This follows from paleogenomic data showing that the Upper Paleolithic populations of Siberia and Eastern Europe (in the time range from 18 to 45.5 thousand years ago) were characterized by approximately the same set of mtDNA haplogroups of West Eurasian origin (e. g., haplogroups N*, U*, U2, U8c, R1b) (Raghavan et al., 2014; Sikora et al., 2017, 2019; Yu et al., 2020). Thus, it is not excluded that the mtDNA X-haplotypes, which emerged on the basis of haplogroup X2a, were also common in the gene pool of this ancient population. The age of haplogroup X2a is approximately 13,000 years (Behar et al., 2012), and its range is limited to northwestern North America only (Hooshiar Kashani et al., 2012).

Haplogroup C4c has a similar geographic distribution and evolutionary age (~14 thousand years) (Hooshiar Kashani et al., 2012; Achilli et al., 2013). This appears to indicate that the carriers of haplogroups X2a and C4c were among the last Beringians who populated North America. Around the same time, but along the Pacific coast, haplogroup D4h3a presumably spread to the south of America (Perego et al., 2009). Thus, the results of mtDNA polymorphism studies indicate that both pathways (both the “Canadian Corridor” and the Pacific Coast of America) may have been used in the settlement of the Americas.

The results of studies of Y-chromosome variability also showed that the male gene pool of Native Americans is characterized by a small set of genetic lineages – these are haplogroups Q-M3, Q-M848 and Q-Z780, widespread among Native Americans, as well as the more rare haplogroups C-P39 and Q-Y4276, typical of Native North Americans, and haplogroup C-MPB373, found in Native South Americans (Pinotti et al., 2019; Colombo et al., 2022). The evolutionary age of these Y-haplogroups ranges from 15.0 to 21.6 thousand years, indicating that they originated in Beringia. This hypothesis is supported by the discovery of the rare haplogroup Q-L804, related to the Native American haplogroup Q-M3, in populations of Northwestern Europe (Norwegians, Swedes, and Britons). Since the divergence time between these haplogroups is ~17.3 thousand years, it is assumed that migrations from Beringia took place not only in the direction of America, but also in the opposite direction to Europe (Pinotti et al., 2019).

In addition, it should be noted that according to mtDNA studies, very rare haplogroups C1e and C1f, related to Native American C1 haplogroups (C1b, C1c, and C1d), were found in Icelanders as well as in the Mesolithic population of Karelia (Yuzhnyi Oleniy Island) (Ebenesersdóttir et al., 2011; Der Sarkissian et al., 2014). Thus, these data allow us to believe that in Beringia there was not a brief stop of human populations that migrated from the south, but indeed the Beringian populations, with their own genetic characteristics and unique mtDNA and Y-chromosome haplogroups, were formed there over a long time.

The question about the duration of the Beringian Standstill is also of great interest and is regularly studied. One of the first genetic studies of this problem concluded that the Beringian Standstill can be estimated at ~15,000 years, if we start from the first discoveries of Homo sapiens remains in the Asian part of the Arctic ~30,000 years ago and until the moment when the Beringian expansion to America took place, i. e. ~15,000 years ago according to genetic data (Tamm et al., 2007). M. Raghavan et al. (2015) showed based on the results of a paleogenomic study that the upper boundary of divergence between the ancestors of the Native Americans and East Asians should be considered as ~23 thousand years In that case, the isolation period in Beringia (before leaving for America 15,000 years ago) was ~8,000 years. Estimates of the duration of the Beringian Standstill obtained by using the analysis of ancient mitogenomes demonstrated that, given various kinds of uncertainties, the isolation period of the Beringian population could have ranged from 2.4 to 9.0 thousand years (Llamas et al., 2016).

Such a long stay in Beringia suggests that its population was quite large in number and subdivided within Beringia (Hoffecker et al., 2016; Moreno-Mayar et al., 2018a). It is assumed that the western part of Beringia (Chukotka) was inhabited by Paleosiberian populations, while the eastern part of Beringia (Alaska and Yukon) was inhabited in the south by the Native American ancestors and in the north by a separate group of ancient Beringians who disappeared or were assimilated by northern Native American tribes (Moreno-Mayar et al., 2018a; Sikora et al., 2019; Willerslev, Meltzer, 2021). Both of these groups of the ancient Eastern Beringians were very closely related, since they showed the C1b and B2 mitochondrial haplogroups typical of the Native Americans (Tackney et al., 2015; Moreno-Mayar et al., 2018b). Moreover, at present these haplogroups are more characteristic of the southern Native American populations, but in the past they were present in the north of Eastern Beringia, thus confirming the Beringian Standstill hypothesis (Tackney et al., 2015; Moreno-Mayar et al., 2018a).

It is likely that during the glacial maximum, the southern part of Beringia was a refugium, characterized by a fairly mild climate, rich biota, and therefore quite suitable for settlement of ancient people (Hoffecker et al., 2016; Sikora et al., 2019). According to the results of paleogenomic data modeling, the divergence between the Paleosiberians and the population that gave rise to the Beringians occurred approximately 24 thousand years ago, but the Upper Paleolithic genetic component was obtained by both populations approximately 20 thousand years ago (in the ancient Beringians its fraction was ~18 %) (Sikora et al., 2019). Thus, it is assumed that the most ancient Upper Paleolithic peoples were actually present in Beringia during the last glacial maximum, and that the formation of Beringians, including Native American ancestors, began through interaction with the newcomer East Asia-related peoples ~20 thousand years ago (Sikora et al., 2019). In this scenario, the time of existence of the relatively isolated Beringian population is estimated to be ~5,000 years.

A study of Y-chromosome polymorphism in Native American populations also showed that the duration of the Beringian Standstill may not have exceeded 4,600 years (Pinotti et al., 2019). Thus, the results of genomic studies suggest that the adaptation to the Arctic conditions of the ancient populations that took refuge in Beringia may have taken several millennia.

## On adaptive selection in Native American ancestors

According to some genetic studies, it is proposed that the genomes of the Native Americans may contain signals of adaptation to the Arctic conditions, which emerged during the Beringian stage of the Native American ancestors formation, which, as noted above, may have taken a fairly long period of time (Ruiz-Pesini et al., 2004; Amorim et al., 2017). A recent study of genomic polymorphism in various contemporary continental groups showed that Native Americans harbor at least 20,424 genetic variants suspected to have originated in Beringia (Niedbalski, Long, 2022). This is comparable to the number of genetic variants that distinguish Africans from non-Africans. However, there are tens of times less specific genetic variants in Eurasia. At the same time, group-specific polymorphisms have not been found at all in Europeans, thus indicating rather intensive inter-ethnic contacts within Eurasia (Niedbalski, Long, 2022).

A study of the influence of adaptive selection on the genetic profile of Native Americans has shown that the appearance of American-specific genetic variants is associated with various metabolic pathways, but the most important ones are associated with the production of melanin in the skin, hair and eyes, as well as cardiovascular function (Niedbalski, Long, 2022).

It should be noted, however, that the most distinct traces of adaptation to the severe conditions of the Far North have been revealed in the gene pools of the Eskimo and Paleo-Asiatic peoples rather than in those of the Native Americans. Eskimo and Paleo-Asiatic populations also originated in the Asian and North American Arctic during the last 5–6 thousand years (Flegontov et al., 2019; Willerslev, Meltzer, 2021) and thus, their periods of adaptation are quite comparable in time to the Beringian population. However, in the next 15 thousand years after their isolation, the Beringians began to occupy more southern American territories, and therefore it is unknown to what extent the traces of their ancestors’ adaptation to the Arctic are preserved in the gene pools of modern Native Americans (Adhikari et al., 2019).

A number of studies of modern and ancient populations of the Far North of Asia and America have found evidence of genetic and physiological adaptation of Eskimo and Paleo- Asiatic ancestors to low temperatures and an “Arctic” diet based primarily on the consumption of seafood rich in polyunsaturated fatty acids (PUFAs) (Cardona et al., 2014; Clemente et al., 2014; Fumagalli et al., 2015).

One of the most striking examples of diet-related genetic markers is the “Arctic” variant of the CPT1A gene (rs80356779-A) (Clemente et al., 2014; Malyarchuk, 2020). It is known that the “Arctic” variant is widely spread in modern populations of the Eskimos, Chukchi, Koryaks, and other peoples of the Sea of Okhotsk region, whose economic style is connected with sea hunting (Malyarchuk et al., 2016). According to paleogenomic data, the earliest records of the “Arctic” variant of the CPT1A gene were found among the Greenland and Canadian Paleo-Eskimos (4,000 years ago), representatives of the Tokarev culture of the North of the Sea of Okhotsk region (3,000 years ago) and carriers of the Late Jomon culture of Hokkaido Island (3,500–3,800 years ago). The appearance of the “Arctic” variant of the CPT1A gene is most likely associated with the adaptive response of the Far North indigenous peoples to the “Arctic” diet, which is characterized by a marked excess of lipids and proteins and a deficit of carbohydrates

In addition, genetic variants associated with carbohydrate metabolism are very frequent in indigenous populations of Northeast Asia. For example, the maximum frequency (52 %) of deletion of the pancreatic amylase gene AMY2A, necessary for starch digestion, was found in the Eskimo, Chukchi, and Koryak populations; ~30 % of the indigenous peoples of Northeast Asia lack this gene (Inchley et al., 2016). The high frequency of AMY2A gene deletion in Northeast Asia may be explained by the deficiency of starch and disaccharides in the traditional diet of indigenous peoples in the past. A deficiency in oligosaccharides can also explain the high incidence of the inactive sucrose-isomaltase gene (gene SI ) among the indigenous populations of the Far North of Asia and America (Malyarchuk et al., 2017; Pedersen et al., 2017). In Greenlandic Eskimos, glucose homeostasis-related mutations in the TBC1D4 and ADCY3 genes have also been identified, which increase the risk of obesity and type 2 diabetes (Moltke et al., 2014; Grarup et al., 2018).

Thus, genetic changes that occurred in the past due to adaptation to extreme environments, in the current conditions (when there is no carbohydrate deficiency in the diets of the Far North indigenous populations) become harmful, because they increase the risk of metabolic and other diseases.

Interestingly, in a number of cases, adaptive changes in the gene pools of Eskimo and Paleo-Asiatic populations are caused by nonsynonymous substitutions with high pathogenicity indices (in the CPT1A, CRAT genes), nonsense mutations leading to stop codons or splicing disruption (in the SI, TBC1D4 and ADCY3 genes), and whole gene deletions (as in the case of the AMY2A gene) (Malyarchuk, Derenko, 2017; Pedersen et al., 2017; Malyarchuk, 2018). It seems likely that this kind of fairly profound genetic changes could have been preserved in the gene pools of Native Americans if they had adapted to the Arctic environment in a similar way, involving a very specific “Arctic” diet.

There is a suggestion that since the ancient inhabitants of Beringia migrated first to the sub-Arctic Pacific coast and then on to Northwest America, they may have practiced a maritime type of economy at the sub-Arctic stage (Hoffecker et al., 2016; Pedersen et al., 2016; McLaren et al., 2018). But, apparently, this stage of migration history was not reflected in the gene pools of the Native Americans, or their ancestors did not hunt sea mammals, but hunted mainly megafauna: mammoths, bison, horses and other terrestrial animals of the Great Arctic Plain (Pitulko et al., 2017; Lindgren et al., 2018; Harris et al., 2019). In that case, it is assumed that Amerindian ancestors had to adapt primarily to vitamin D deficiency, because at high latitudes UV radiation levels are insufficient for annual synthesis of cholecalciferol D3 in the skin, and the intake of ergocalciferol D2 depends on diet

Genetic adaptation to vitamin D deficiency suggests that the dietary sources of this substance that are available to Arctic peoples (e. g., sea mammals, fish, eggs) were scarce or absent in the Beringian steppe-tundra biome. Vitamin D deficiency affects the health of children to a greater extent, and so L.J. Hlusko et al. (2018) suggested that Beringians adapted to UV deficiency through a V370A substitution in the ectodyslasin receptor encoded by the EDAR gene. This mutation is widespread in populations of East and North Asia, as well as in Native Americans. One manifestation of this mutation is associated with an increase in the branching density of mammary gland ducts, which leads to higher amounts of vitamin D in breast milk (Hlusko et al., 2018).

A recent study of Latin American populations also showed that genes related to energy metabolism played a crucial role in population adaptation to the environment of America (Mendoza-Revilla et al., 2022). The great importance of immune response genes, primarily HLA genes, in adaptive processes has also been highlighted. Moreover, positive selection in these genes is recorded in Native Americans at different periods of their history, both after the European colonization of America, which introduced new pathogens, and during the initial settlement of America at the first contact with endemic pathogens (Lindo et al., 2016; Mendoza-Revilla et al., 2022).

## FADS gene cluster

The hypothesis suggesting the influence of high-latitude UV deficiency on the genetic features of the ancient Beringians is based on an additional argument involving the adaptive selection of the FADS genes variants in Native Americans (Hlusko et al., 2018). The fatty acid desaturase genes FADS1 and FADS2 encode enzymes involved in PUFA biosynthesis from shorter precursors (Nakamura, Nara, 2004). It is assumed that the ancestral A-variants of the FADS genes gave advantages to human populations that consumed food rich in lipids and proteins (for example, the Upper Paleolithic population of Eurasia), but later in the Neolithic, after the emergence of agricultural technology, the D-variants became more common, allowing for a higher rate of synthesis of PUFAs from plant lipids (Ameur et al., 2012; Mathieson S., Mathieson I., 2018).

Studies have shown that FADS genes have been affected by positive selection for a long time – for example, in Europe there has been an increase in the frequency of haplotype D from less than 10 % 10,000 years ago to 60–75 % at present (Mathieson, 2020). However, the exact reasons for the increased frequencies of the FADS genes variants in certain regions of the world have not yet been established. This is due to the fact that FADS genes are highly pleiotropic. Associations were found for FADS1 and FADS2 genetic variants with blood lipids and other metabolites, various blood cell phenotypes, suggesting a link with cardiovascular function (Draisma et al., 2015). Besides, metabolic balance with respect to PUFAs and other lipids is very important for brain function, in which the FADS1 gene is actively expressed (Mathieson et al., 2020)

It has been suggested that the emergence of the D haplotype may have been due not only to the need to increase the efficiency of PUFA synthesis when there is a deficiency in the diet (for example, in Africa, where the frequency of the D haplotype is still high), but also contributed to an increase in the brain size (Ameur et al., 2012). In addition, it was found that in Greenlandic Eskimos, FADS genes are involved in the processes of adaptation to the cold, as there are links between genetic variants and the distribution of body fat and height; and the participation of FADS genes in the regulation of growth hormones is also suggested (Fumagalli et al., 2015).

Population genetic studies have shown that the ancestral haplotype A is much more common among the indigenous populations of Siberia and America (Amorim et al., 2017; Hsieh et al., 2017; Ye et al., 2017; Malyarchuk, Derenko, 2018). It has also been found that in Greenlandic Eskimos and Native Americans, the FADS1 and FADS2 genes are under positive selection, which contributed to an almost complete fixation of haplotype A in these populations (Fumagalli et al., 2015; Amorim et al., 2017). Meanwhile, a number of other studies on Arctic populations of Siberia (Eskimos, Chukchi, Koryaks, Nganasans, Yakuts) and North America (Eskimos) have not revealed the effect of selection on the FADS1 and FADS2 genes (Cardona et al., 2014; Hsieh et al., 2017; Reynolds et al., 2019).

Nevertheless, there is a hypothesis that the original positive selection signal in the FADS1 and FADS2 genes arose in the ancient population of Beringia due to the need to adapt to the cold and limited food resources provided by the Arctic (Amorim et al., 2017). It is assumed that these adaptive genetic variants have been preserved in the gene pools of the Native Americans and Eskimos. However, different combinations of the FADS genes variants are responsible for the effect of selection in Arctic and Native American aborigines and in Europeans (Fumagalli et al., 2015; Amorim et al., 2017; Ye et al., 2017; Mathieson, 2020). This can be due to different reasons for the selection of the FADS genes variants in populations. In Europeans it was a shift towards a plant-based diet in the Neolithic, due to which the D-haplotypes with increased desaturase activity were more favorable, and in Eskimos ancestors it was the emergence of a specific “Arctic” diet with very high PUFA content, due to which maintaining the maximum frequency of A-haplotypes was more preferable. It is possible that these FADS variants in Eskimos possess even lower desaturase activity (Mathieson, 2020).

Meanwhile, a recent analysis of the FADS genes polymorphism in modern and ancient populations has shown that the widespread distribution of haplotype A occurred under the influence of selection already in the Upper Paleolithic populations of Eurasia, and therefore in the gene pool of the ancient Beringians this haplotype could be fixed quite accidentally under the effects of genetic drift (Harris et al., 2019; Mathieson, 2020). Thus, the adaptive changes of FADS genes in the Native American ancestors are questioned, and the hypothesis that the FADS haplotypes inherited from the Upper Paleolithic Eurasians are preserved in the gene pools of Native Americans looks more likely

## ACTN3 gene

According to the published data, another example of selective advantages obtained by Native Americans from the ancient inhabitants of Beringia may be related to adaptive changes in the ACTN3 gene (Amorim et al., 2015). This gene encodes the protein α-actinin-3, which is expressed exclusively in fast-twitch skeletal muscle fibers. Of the polymorphic loci of the ACTN3 gene, the most studied is rs1815739, a mutation in which leads to termination of protein synthesis at amino acid position 577 of exon 16 (R577X substitution) (North et al., 1999). This causes a deficiency of α-actinin-3 in fasttwitch skeletal muscle fibers, which, in turn, can result in a decrease in the speed and power indicators of human physical performance (Alfred et al., 2011). Genetic studies have shown that the frequency of the 577X allele increases with distance from Africa to the north of Eurasia and reaches a maximum in the Native American populations (Amorim et al., 2015). It is suggested that the advantages of the 577X allele, such as increased endurance and improved protection against the cold, have contributed to the spread of this mutation (Bramble, Lieberman, 2004; Wyckelsma et al., 2021).

Recent physiological and metabolomic studies have shown that carriers of the 577XX genotype tolerate cold much better than 577RR individuals (Wyckelsma et al., 2021), which may support the hypothesis of selection of the X allele at the initial stage of human expansion into Eurasia (Amorim et al., 2015). According to this hypothesis, the elevated frequency of the 577X allele in some human populations could be related to selection to improve metabolic efficiency and promote dynamic activities (e. g., long hunting and, in sports, marathon running, swimming, cycling).

The high frequency of the 577X allele in Native Americans suggests that this allele was also widespread in ancient Beringians, ancestral to Native Americans. However, the results of analysis of the distribution of the rs1815739 polymorphic variants in the indigenous populations of northeastern Siberia (in Chukchi, Koryaks, and Evens) have shown that the frequencies of the 577X allele and 577XX genotype were not the highest in Eurasia (Malyarchuk et al., 2018). While the frequency of the 577X allele in Northeast Siberia is approximately 36 % (Malyarchuk et al., 2018), the maximum frequencies of this genetic variant are registered in South Asian and Native American populations – over 60 % according to dbSNP database (www.ncbi.nlm.nih.gov/snp).

A recent analysis of geographic distribution of rs1815739 variants in modern and ancient populations has shown that population frequencies of the 577X allele correlate neither with geographic latitude nor with temperature (Mörseburg et al., 2022). None of the statistical tests used in this study found evidence of the effect of positive selection for the 577X allele at high latitudes. Thus, the high frequency of this genetic variant in Native Americans is most consistent with the effect of genetic drift, possibly occurring in Beringia at the stage of low size of ancestral population. It is possible, however, that some advantages of this genetic variant, namely increased endurance and resistance to cold, contributed to the increased frequency of the 577X allele in Native American ancestors.

## Conclusion

The results of genetic studies of modern and ancient populations of Eurasia and America have demonstrated quite convincingly that the role of Beringia in the settlement of the Americas is very high. To date, sufficiently reliable estimates of divergence times between the ancestral genetic lineages that led to the formation of the various populations of Northeast Asia and the Americas have been obtained. The most probable scenario seems to be that during the last glacial maximum, the population that gave rise to the Native Americans ancestors persisted in Beringia for several millennia (20,000–15,000 years ago). It is assumed that this was a relatively small (from several hundred to several thousand individuals (Fagundes et al., 2018)) group of people well adapted to high latitudes and cold environments, which is confirmed by the results of genomic studies of modern Native American populations.

Possible traces of adaptation to Arctic environments at the genomic level are associated with various metabolic pathways – with melanin synthesis processes, cardiovascular system functioning, energy metabolism, and immune response genes (Mendoza-Revilla et al., 2022; Niedbalski, Long, 2022). It has also been suggested that the relaxation of negative selection in a number of protein-coding genes observed in Native Americans is also associated with the Beringian stage of population adaptation (Niedbalski, Long, 2022). However, it is still unclear to what extent such adaptive signals could have survived, given the approximately 15-thousand-year period of American colonization following the Beringian Standstill (Adhikari et al., 2019).

Obviously, answers to such questions can be obtained by using paleogenomic data, but very few ancient human remains have been found from the territory of ancient Beringia. So far, we can get some insight into the genetic features of the Beringians from only one genome of the Eastern Beringian descendant (11.5 thousand years ago, Upward Sun River site, Alaska) (Moreno-Mayar et al., 2018a). Thus, investigations into the role of Beringia in the origins of the Native American ancestors remain relevant and ongoing.

## Conflict of interest

The authors declare no conflict of interest.
